# Ether *v.* Chloroform

**Published:** 1902-06-14

**Authors:** 


### ETHER *v.* CHLOROFORM.

In the discussions which so frequently arise in regard to the comparative safety of ether and chloroform when given as anæsthetics, one great cause of uncertainty is the fact that it is only by dealing with large numbers that useful results can be obtained, while when large numbers are collected from different authorities and institutions there is always the chance of many errors. Mr. Roger Willams,[1] however, points out that by appealing to the well-kept records of the administration of anæsthetics as tabulated in the St. Bartholomew's Hospital Reports, which go back as far as 1875, one may obtain reliable data. The results of such an appeal are sufficiently striking. During the 16 years, 1875–1890, chloroform was administered 19,526 times with 13 deaths, and during the 10 years from 1891–1900 it has been given 23,452 times with 20 deaths. Thus, during the 26 years 1875–1900 chloroform was administerd 42,978 times with 33 fatalities, or 1 in 1,300. With ether the result was very different. During the 16 years 1875 to 1890 ether was given 21,332 times with only 4 deaths. In 12,941 of these cases ether was preceded by "gas," and in this category there was only one fatality, while in the other 8,391 cases in which ether alone was used three deaths occurred. During the 10 years 1891–1900 ether alone has been given 4,826 times, and ether preceded by "gas" 15,945 times and no death has occurred at all. Thus, during the 26 years 1875–1900 ether was administered 37,277 times with only four fatalities, or 1 in 9,319. It follows, says Mr. Roger Williams, that ether is a very much safer anæsthetic than chloroform (in the proportion of 1,300 to 9,319), and that much safer than either of these agents alone is ether preceded by "gas."

[1] Lancet, June 7.

